# Enhancing Mechanical and Tribological Properties of Epoxy Composites with Ultrasonication Exfoliated MoS_2_: Impact of Low Filler Loading on Wear Performance and Tribofilm Formation

**DOI:** 10.3390/nano14211744

**Published:** 2024-10-30

**Authors:** Ravisrini Jayasinghe, Maximiano Ramos, Ashveen Nand, Maziar Ramezani

**Affiliations:** 1 Department of Mechanical Engineering, Auckland University of Technology, Auckland 1010, New Zealand; ravisrini.jayasinghe@autuni.ac.nz (R.J.); maximiano.ramos@aut.ac.nz (M.R.); 2 Faculty of Engineering, University of Auckland, Auckland 1010, New Zealand; ashveen.nand@auckland.ac.nz

**Keywords:** coefficient of friction, epoxy composite, high intensity ultrasonication, molybdenum disulfide solid lubricant, wear rate

## Abstract

This study highlights the impact of low amounts of MoS_2_ quantities on composite performance by examining the effects of ultrasonication exfoliated MoS_2_ at different loadings (0.1–0.5 wt%) on the mechanical and tribological parameters of epoxy composites. Even at low concentrations, the ultrasonication and exfoliation procedures greatly improve the dispersion of MoS_2_ in the epoxy matrix, enabling its efficient incorporation into the tribofilm during sliding. Optimum mechanical properties were demonstrated by the MoS_2_/epoxy composite at 0.3 wt%, including a modulus of elasticity of 0.86 GPa, an ultimate tensile strength of 61.88 MPa, and a hardness of 88.0 Shore D, representing improvements of 61.5%, 35.45%, and 16.21%, respectively. Corresponding tribological tests revealed that high sliding velocity (10 N load, 0.2 m/s) resulted in a 44.07% reduction in the coefficient of friction and an 86.29% reduction in wear rate compared to neat epoxy. The enhanced tribological performance is attributed to the efficient removal and incorporation of MoS_2_ into the tribofilm, where it acts as a solid lubricant that significantly reduces friction and wear. Even though an ultra-low amount of filler concentration was added to the composite, a unique finding was the high MoS_2_ content in the tribofilm at higher sliding speeds, enhancing lubrication and wear protection. This study establishes that even ultralow MoS_2_ content, when uniformly dispersed, can profoundly improve the mechanical and tribological properties of epoxy composites, offering a novel approach to enhancing wear resistance.

## 1. Introduction

Epoxy resins are widely recognized for their ability to work seamlessly with a diverse range of reinforcement materials, providing outstanding mechanical, thermal, and chemical properties, along with considerable design versatility. Because of these exceptional attributes, epoxy resin remains a top choice for many high-performance technical uses [1,2]. However, despite its impressive performance, there is still room for improvement in enhancing both the performance and durability of epoxy composites. Epoxy resins find widespread application in the fabrication of glass or carbon fiber laminates, as well as in the incorporation of fibrous or particle fillers to create advanced, multifunctional composite materials requiring high specific strength. While epoxy offers many desirable properties for various applications, its inherent brittleness can compromise its mechanical properties. Additionally, epoxy has limited fracture toughness and low wear resistance, further constraining its suitability for different applications, including tribology [2,3].

In recent years, improved polymer composites containing reinforcing filler and solid lubricants have gained use in self-lubricating components designed to perform without external lubrication in tribology applications [4,5]. The fundamental purpose of solid lubricant materials used to create the self-lubricating composites or coatings in tribology is to mitigate friction and wear in machinery subjected to sliding and rotating motions. Apart from lubricity, an excellent solid lubricant material should possess specific attributes, including high resistance to oxidation, exceptional thermal and chemical stability, thermal conductivity, and low shear strength, that meet the demands of various engineering applications [6]. The manufacturing of self-lubricating bearings is a common usage of self-lubricating materials in the automotive, aviation, power, electronics, and domestic appliance sectors. Molybdenum disulfide (MoS_2_) is widely recognized as an effective oil-based nanofluid additive due to its unique lubricating properties. When dispersed in oil, MoS_2_ nanoparticles act as solid lubricants, reducing friction and wear by forming a protective tribofilm on the contact surfaces during operation. The layered structure of MoS_2_ allows easy sliding between layers, contributing to its exceptional lubricity. Additionally, MoS_2_ nanoparticles enhance the load-carrying capacity and thermal stability of the lubricant, improving performance under high-pressure and high-temperature conditions. This mechanism leads to smoother operation, reduced energy consumption, and extended life of mechanical components in various industrial applications [5,7]. However, the nonlinearity of the thermal expansion coefficient, the creep phenomena common to polymers, softness at high temperatures, shrinkage during processing, and the humidity causing swelling are some limitations and restrictions on the use of polymers as a composite matrix material for self-lubricating applications [7].

Therefore, it is necessary to consider several major factors in fabricating self-lubricating composites using polymers for applications such as bearings, gears, cams, and brakes. The main consideration when incorporating self-lubricating additives into polymer composites is to reduce the wear rate and friction coefficient while sliding. However, improving the polymer’s load-carrying ability is also a major consideration in composite development for load-bearing applications. Polymer composites can be reinforced with fibers or particles, although fiber reinforcement often has a higher load-carrying capacity than particles. However, since lubricating additives can simply slide along the planes and reduce the load-carrying performance, one must use them with precaution while using them. This is particularly true when using specific inorganic solid lubricating additives. Therefore, self-lubricating composites are frequently made with both lubricating particles and reinforcing fibers, or occasionally with a combination of non-lubricating and lubricating fibers, when better lubrication and a higher load-carrying capacity are required. Moreover, improving the thermal conductivity of the composite is also crucial since sliding material buildup heat and typically polymers have limited heat conductivity [8].

The following classes of compounds are widely considered as solid lubricants for use in the self-lubricating polymer composites: soft metals, layered materials (graphite, hexagonal boron nitride, and transition metal dichalcogenides), alkaline earth fluorides, binary oxides (PbO, B_2_O_3_, Magnéli phases TiO_2_, and V_2_O_5_), and oxides (molybdates, tungstates, vanadates, tantalates, chromates, sulfates, and silicates), oxythiomolybdates, and MAX phases (Ti_3_SiC_2_, Ti_2_AlC, etc.) [9]. Two-dimensional nanomaterials, like graphene, MoS_2_, black phosphorus (BP), etc., have layered structures and are easily sheared and ideal for enhancing the ability to reduce friction and resist wear [10]. MoS_2_ has lubricating qualities that stem from its lamellar crystalline structure, which is made up of weak Van der Waals forces that hold the sulfur lamellar together. This arrangement facilitates shear, allowing the MoS_2_ layers to slide easily and align parallel to the direction of relative movement, thereby providing lubrication. Additionally, the strong ionic bond between sulfur and molybdenum provides a high resistance to surface irregularities in the lamella structure [11]. However, MoS_2_’s inherent lubricating ability is sensitive to oxygen and water vapor absorption from the air, which results in a restricted amount of lubrication in the atmosphere. MoS_2_ is therefore frequently utilized in aerospace applications and other vacuum environment situations [12].

In recent years, scientists have gained attention for the use of MoS_2_ powder as an additive material for developing epoxy composites in a variety of applications. Madeshwaran et al. [13] researched the effects of MoS_2_ on the thermal expansion and mechanical properties of epoxy composites. They utilized MoS_2_ nanosheets obtained through ultrasonication exfoliation and incorporated them into epoxy as nanofillers through mechanical stirring. Their results indicate a significant reduction in the composites’ thermal expansion coefficient with the addition of exfoliated MoS_2_. Moreover, the incorporation of an ultralow content (0.1% by weight) of MoS_2_ filler in the epoxy matrix led to a significant increase in both flexural (9.0%) and tensile strength (39.2%) properties of the composites. This enhancement in the mechanical properties of epoxy nanocomposites can be attributed to the substantial interfacial bonding between epoxy and MoS_2_. Several studies have been reported in the literature to determine the enhancement of properties of epoxy composites by incorporating MoS_2_. Zhao et al. [14] used carbon nanotubes (CNTs) wrapped with MoS_2_ nanolayers to make a hybrid MoS_2_-CNT that enhanced the thermal stability, flame retardancy, and mechanical properties of epoxy. Xia et al. [15] utilized a solution attributed to the barrier effect of MoS_2_ nanosheets, which reduced the diffusion of corrosive agents. This suggests that MoS_2_ nanosheets contribute to enhancing the mechanical properties of SiO_2_-MoS_2_ composite coatings by improving interfacial interaction. Ji et al. [16] studied the synergistic effect of (CNT)/MoS_2_/graphene in an epoxy matrix to enhance the thermal conductivity of the composite. They found that MoS_2_ sheets, possessing a large specific surface area, efficiently gather heat from the epoxy matrix, thus promoting effective heat dispersion, which is beneficial for using MoS_2_ as a solid lubricant.

Few studies have explored the utilization of MoS_2_’s self-lubricating properties within an epoxy matrix. Li et al. [17] designed an epoxy composite with graphite/MoS_2_ as lubricating coatings for switch slide baseplates in railway applications. Their findings revealed that graphite in the epoxy composite effectively reduced the friction coefficient and significantly enhanced the wear resistance of the composite. During wear tests, MoS_2_ in the epoxy composite transformed into MoO_3_. As a result, the friction coefficient of the pair could not be reduced since MoO_3_ lacks a layered structure. However, MoS_2_ effectively improved the wear resistance of the composite by inhibiting the transfer of epoxy from the composite’s surface to the surface of A36 steel. Liu et al. [18] investigated the combined impact of incorporating modified SiC and MoS_2_ into epoxy using the silane coupling agent KH560 on wear and friction properties. They observed that adding an appropriate amount of MoS_2_ resulted in its uniform dispersion throughout the material. During friction, the MoS_2_ gradually migrated to the surface, forming a lubricating film that improved the smoothness of the worn surface. However, increasing the content of hard abrasive SiC particles on the wear surface caused slight abrasive wear and elevated both the friction coefficient and volume wear rate. Moreover, exceeding the load capacity of the modified MoS_2_/SiC/EP composite resulted in partial destruction of the lubricant film, with fatigue wear becoming the primary wear mechanism. Upadhyay and Kumar [19] examined a ternary composite comprising epoxy, graphene, and MoS_2_, analyzing their physiochemical, thermal, and tribological characteristics. They discovered that the inclusion of graphene and MoS_2_ in the epoxy matrix improved tribological behavior by mitigating adverse wear mechanisms. In the presence of air or oxygen, MoS_2_ can generate sulfides and oxides, which negatively impact tribological properties. However, the addition of graphene to the polymer matrix reduces the occurrence of these harmful reactions, resulting in reduced friction and wear. Moreover, graphene exhibits robustness under sliding contact and serves as a lubrication mechanism without degradation. Previous research has highlighted that the strong van der Waals forces between MoS_2_ sheets, along with their large specific surface area, facilitate the easy agglomeration of exfoliated MoS_2_ nanosheets in the matrix [14]. Additionally, the absence of reactive groups on the surface of MoS_2_ layers makes it challenging for organic molecules or monomers to be grafted on them, thus directly limiting the utility of MoS_2_ in polymer composites [15].

Among the few studies that have been previously published, it is indicated that the MoS_2_ lubricating effect predominated when combined with the epoxy composite’s synergistic effects of graphite, graphene, or SiC. According to Liu et al. [18], a manual mix of 4 wt.% of MoS_2_ with 55 wt.% SiC reduced the friction coefficient and volume wear rate by 10.06% and 52.13%, respectively, under test conditions with a load of 50 N, a rotation rate of 200 rpm, and a total wear distance of 5000 m. The study by Upadhyay and Kumar [19] showed that the average friction coefficients of binary neat epoxy, epoxy-graphene, epoxy-MoS_2_, and ternary epoxy-graphene-MoS_2_ composites that were mixed by mechanical stirring are in the range of 0.30–0.33, 0.21–0.24, 0.14–0.15, and 0.23–0.48, respectively, with an applied load of 1–2 N and a rotation speed of 1000–3000 rpm. Further, they mentioned that a 10 wt.% graphene10 wt.% MoS_2_ composite weight ratio gives the lowest COF values. Therefore, it is worth noting that relatively large amounts (10–40 wt%) of MoS_2_ material quantities are often necessary for better tribological modification. However, this can lead to sedimentation, clumping, higher viscosity, or unnecessary cost increases, which are significant drawbacks that require careful consideration in bulk-level industrial manufacturing.

Epoxy composites are integral in engineering applications due to their versatility and strong mechanical properties. However, their inherent brittleness and limited wear resistance restrict their use in high-performance scenarios. Traditional methods to enhance these properties often involve adding significant amounts of fillers, which can compromise the material’s integrity.

## 2. Materials and Methods

### 2.1. Materials

Bisphenol A/epichlorohydrin 50–70 wt% epoxy resin, Isophoronediamine (3-aminomethly-3,5,5-trimethyl-cyclohexylamine) 50–60 wt% curing agent, and the mold release agent were purchased from Norski, Plimmerton, New Zealand. Molybdenum (IV) sulfide with a flaky morphology, having a thickness of several microns, grain size less than 2 μm, and purity >98% was obtained from AK Scientific in San Francisco, CA, USA. Ethanol (C_2_H_5_OH) with 99.9% purity was obtained from Merck Life Science Ltd., Auckland, New Zealand.

### 2.2. Methodology

First, 800 mg of flaky MoS_2_ powder was measured and placed in a 1 L beaker. Next, the powder was dispersed in a mixture of 45% ethanol and 55% water, totaling 500 mL. It was then sonicated for 20 min by Sonics Materials VC-505–220, Vibra Cell, high-intensity ultrasonicator (12 mm titanium tip of a 20 kHz ultrasonic horn transducer with a nominal power of 550 W and a nominal output frequency of 20 kHz). The process was repeated up to 5 times. Following sonication, the mixture was centrifuged at 5000 rpm for 15 min. The resulting supernatant was collected, dried, and weighted, and then the MoS_2_ samples were prepared from it for studying their microstructure by SEM.

The MoS_2_/epoxy composite was manufactured by an open mold process, utilizing MoS_2_ as both a reinforcement and a self-lubricating filler, while epoxy served as the matrix. Standard precautions were taken to ensure precise mixing and homogeneity of the epoxy formulation. Exfoliated layers of MoS_2_ powder were carefully measured and added to the epoxy resin. The MoS_2_/epoxy composites with a mass fraction of MoS_2_ of 0.1, 0.2, 0.3, 0.4, and 0.5 wt% were obtained, respectively. The mixture was then mechanically stirred for one hour to achieve a uniform blend of epoxy and MoS_2_ powder. Next, the dispersion of MoS_2_ layers in the epoxy resin was further accomplished using the high-intensity ultrasonic horn mentioned above. The ultrasonic horn was submerged into the liquid MoS_2_/epoxy resin mixture, with the beaker placed in an ice water bath to prevent excess heat generation. The parameters of the ultrasonic dispersion process were kept the same as exfoliation to ensure optimal dispersion.

Next, the curing agent, isophoronediamine, was added to achieve a 4:1 ratio of epoxy to hardener at room temperature. Following this, the mixture underwent degassing in a vacuum chamber under −1 bar pressure for 10 min to eliminate trapped air. Subsequently, the prepared mixtures comprising epoxy resin, MoS_2_, and hardener were poured into pre-prepared molds. The surfaces of the aluminum mold were smoothed using a scotch pad to ensure a smooth, even, and uniform molding surface. A mold release agent was applied, and sufficient time was allowed to dry at room temperature. The curing time and temperature were determined based on recommendations from the epoxy datasheet given by the supplier. The curing process involved initial curing overnight at temperatures as low as 10 °C, followed by post-curing overnight at 60 °C to enhance the ultimate strength of the composite. This cyclic curing process facilitated proper crosslinking and hardening, resulting in the formation of solid and composite specimens ready for subsequent testing and characterization. The determination of the cure cycle is critical for the successful fabrication of epoxy composite products with reliable quality and cost-effectiveness. The non-linear increase in internal temperature caused by the exothermic chemical reaction of epoxy can lead to temperature overshoot. Non-uniform curing can result in incomplete cure, resin degradation, and the entrapment of volatiles or voids, ultimately diminishing the overall quality and performance of the finished component [20]. Additionally, a reference sample without any MoS_2_ was fabricated to serve as a baseline for comparison. This reference sample allows for a direct assessment of the influence of MoS_2_ on the mechanical and tribological performance of the epoxy composite.

### 2.3. Test Procedure

The tensile specimens were produced using EN ISO 527-2 type 1b geometry [21]. The specimens were created as dog bone shapes with a width of 4 mm and a length of 150 mm, with a gauge length of 50 mm. The composite samples were tensile tested using a computer-controlled servo-hydraulic universal testing machine. The machine’s maximum load capacity was 50,000 N, and the tension rate was set to 1.5 mm/min. The stress–strain curves obtained were used to derive mechanical properties of ultimate tensile strength, Youngs modulus, and elongation of the epoxy composites according to ISO 527-1 [22]. As per ASTM D2240 [23], a durometer type D was utilized to determine the hardness. The average values were determined for every set of five samples by repeating the same two experiments.

The tribological specimens, designed specifically for tribological testing, were plates of 5 mm in height, 40 mm in length, and 25 mm in width. An Rtec tribometer MFT-5000 (Rtec Instruments, San Jose, CA, USA) was used to conduct linear reciprocating sliding wear tests. The counter material consisted of a chromium steel ball with a diameter of 10 mm and a surface roughness of 2.5 µm. Dry sliding was conducted under controlled test conditions at a constant temperature of 24 °C and a humidity level of 60%. To eliminate any lingering contamination from previous experiments, acetone was utilized to clean both the composite surfaces and the counter ball before each test. Tribological experiments were executed at sliding frequencies of 2 Hz, 5 Hz, and 8 Hz, resulting in sliding speeds of 0.02 ms^−1^, 0.07 ms^−1^, and 0.2 ms^−1^. The applied normal loads were 5 N, 10 N, and 15 N, resulting in 121.73 MPa, 153.39 MPa, and 175.58 MPa maximum applied pressures (calculated using the Hertz ball on flat contact theory) [24]. These test conditions were carefully chosen, including the contact pressure and sliding velocities, which were slightly higher than those used in industrial polymer bearings [25]. The duration of testing ranged from 68 min, 95 min, and 248 min, corresponding to the various sliding speeds, while maintaining a consistent total sliding distance of 400 m. To ensure precision and repeatability, each experimental condition was repeated three times. Subsequently, the average coefficient of friction was computed, taking into consideration the standard errors associated with the measurements. The specific wear rate was calculated using Equation (1):

(1) $$\mathrm{Specific}\mathrm{wear}\mathrm{rate}\;=\;\frac{WSV\;}{W\times L}$$

where *WSV* represents the worn surface volume, *W* is the applied load, and *L* is the total sliding distance. The samples’ wear volume was estimated using a surface profilometer (Taylor Hobson Form Talysurf 50, Leicester, UK) and a diamond stylus (4 µm diameter). The total sliding distance was kept at 400 m, and the wear track width was kept at 7 mm. The raw data from the profilometer reading were processed using MATLAB 2022 to determine the surface track depth, width, and radius, ultimately, the worn surface volume.

Before the tribological test, all samples underwent polishing using 1200 grit papers to stabilize their surface conditions. Measurements of track width and depth were taken using the stylus profilometer to estimate the wear volume of the samples. Following friction testing, wear debris, the steel counterpart, and the wear track on the composite surfaces were inspected using a scanning electron microscope (SEM) (Hitachi SU-70 Schottky field emission scanning electron microscope, Düsseldorf, Germany). The specimens were sputter-coated with platinum (Pt) for 60 s by Hitachi E-1045 ion sputter. The accelerating voltage for the SEM micrograph was 10 kV. The composition of particles was examined by energy dispersive X-ray spectrometry (EDS) with the Noran System 7 (NSS) microanalysis system. The accelerating voltage for EDS was 15 kV.

## 3. Results and Discussion

### 3.1. SEM Observation of Layered MoS_2_

In a single-layered MoS_2_ film, Mo (+4) and S (−2) atoms are organized in an S–Mo–S orientation, with each layer surrounding one Mo atom with six S atoms. MoS_2_ typically exists in two structural phases: trigonal prismatic (2H/3R) or octahedral (1T) [26]. Here, we exfoliated MoS_2_ using an ultrasonication method. Exposing an aqueous suspension of MoS_2_ to ultrasound treatment significantly influences the particle size distribution of MoS_2_. The cavitation phenomenon induced by ultrasound leads to turbulence, resulting in the breakdown of the layered structure of MoS_2_ along the vertical direction. Consequently, the particle size decreases, a consequence of the turbulence and cavitation forces generated by ultrasound treatment [27]. Further, sonication of MoS_2_ in a 45 vol% ethanol/water solution improves the dispersion of the suspension layers. MoS_2_ sheets have been shown to have great solubility when dissolved in a 45 vol% ethanol/water solution [28].

**Figure 1:**
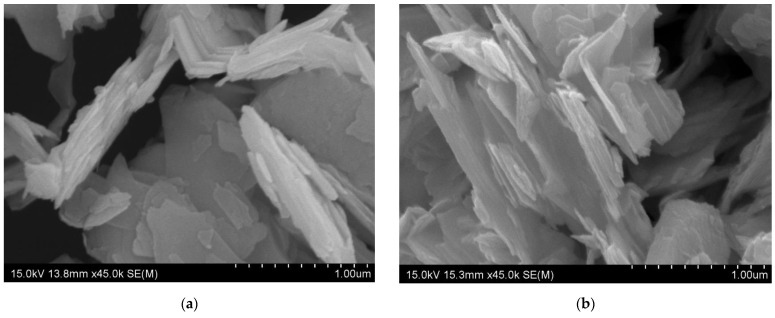
SEM image of (**a**) initial MoS_2_ powder having the layered structure; (**b**) SEM image of exfoliated MoS_2_ powder having the reduced layer thickness.

This reduction in layer thickness simultaneously increases the surface area, rendering MoS_2_ an advantageous filler material. Direct ultrasonication of bulk MoS_2_ in either organic solvents or aqueous surfactant solutions offers a method to prevent alterations in the chemical and electronic characteristics of the MoS_2_ nanosheets [29]. Figure 1a shows SEM observation illustrating the initial MoS_2_ powder with lateral particle sizes ranging from 0.2 to 0.4 μm and grain diameters typically between 2 and 5 microns. MoS_2_ sheets are dispersed more readily in ethanol/water solutions when larger crystallites are broken up into smaller crystallites by subsequent sonication. Following the sonication treatment, the thicknesses of the nanosheets slightly reduced, ranging from 45 to 90 nm, according to SEM imaging Figure 1b.

### 3.2. Tensile and the Hardness Properties of the MoS_2_/Epoxy Composite

Achieving a consistent distribution of fillers in a polymer is a significant challenge when creating epoxy nanocomposites. Agglomeration, which occurs when particles clump together in clusters, can have negative effects on the thermal, mechanical, and tribological properties of the epoxy. This type of clustering does not reflect the desired properties of a polymer composite. For this study, we used the low-viscosity epoxy resin to obtain a uniform distribution of MoS_2_ in epoxy.

**Figure 2:**
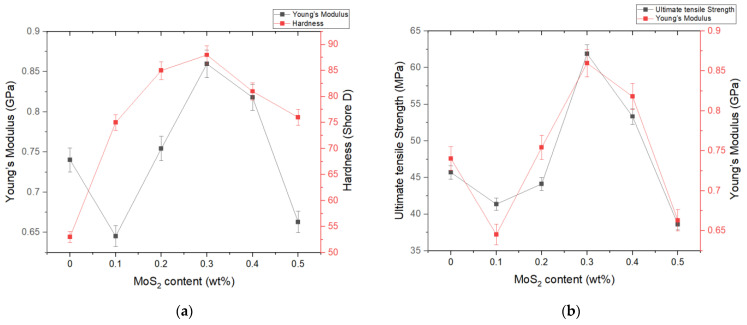
(**a**) Hardnesses and Young’s moduli of composite vary with different MoS_2_ contents; (**b**) Young’s moduli and UTS of composite vary with different MoS_2_ contents.

The tensile test was carried out according to the ISO 527-1 standard, and Table 1 summarizes the results of the tensile tests and the hardness test. Figure 2a shows the changes in hardness and Young’s modulus of neat epoxy and MoS_2_/epoxy composites with varying weight fractions, ranging from 0.1 wt% to 0.5 wt%. The results indicate that both hardness and Young’s modulus increase with increasing MoS_2_ filler content, up to 0.3 wt%. However, a decreasing trend is observed beyond 0.3 wt%. For Young’s modulus, the values for neat epoxy, 0.1 wt% to 0.5 wt% MoS_2_/epoxy composites were 0.74, 0.65, 0.75, 0.86, 0.82, and 0.66 GPa, respectively. This represents a 16.21% increase when adding 0.3 wt% MoS_2_ compared to neat epoxy.

Regarding hardness, the values for neat epoxy and 0.1 wt% to 0.5 wt% MoS_2_/epoxy composites were 53.3, 75.2, 85.1, 88.0, 81.2, and 76.24 Shore D, respectively. This indicates a 65.1% increase compared to neat epoxy when adding a small amount of MoS_2_ (0.3 wt%). The improvements are due to the uniform distribution of MoS_2_ in the epoxy matrix. However, beyond 0.3 wt%, both Young’s modulus and hardness decreased rapidly. These findings suggest that the distribution and exfoliation of MoS_2_ sheets in the epoxy matrix play a crucial role, as noted in prior studies [30].

Figure 2b illustrates the variations in ultimate tensile strength (UTS) and Young’s modulus across filler concentrations ranging from 0.1 wt% to 0.5 wt% MoS_2_/epoxy. While Young’s modulus of the epoxy exhibited insignificant changes upon the addition of MoS_2_ to the matrix, notable improvement was observed in the UTS of the composite compared to neat epoxy. The UTS values for neat epoxy and 0.1 wt% to 0.5 wt% MoS_2_/epoxy composites were 45.68, 41.36, 44.13, 61.88, 53.33, and 38.62 MPa, respectively, representing a 35.45% increase compared to neat epoxy. This enhancement in hardness, UTS, and Young’s modulus at low filler content (0.3 wt%) can be attributed to the effective dispersion of MoS_2_ and strong interfacial interaction between the nanofiller and polymer molecules. However, at higher nanofiller contents (beyond 0.3 wt%), the MoS_2_ sheets become excessively close, leading to agglomeration and restacking due to van der Waals forces [13]. The mechanical test results clearly indicate the reinforcing effect of exfoliated MoS_2_ at a very low filler content, approximately 0.3 wt%.

**Figure 3:**
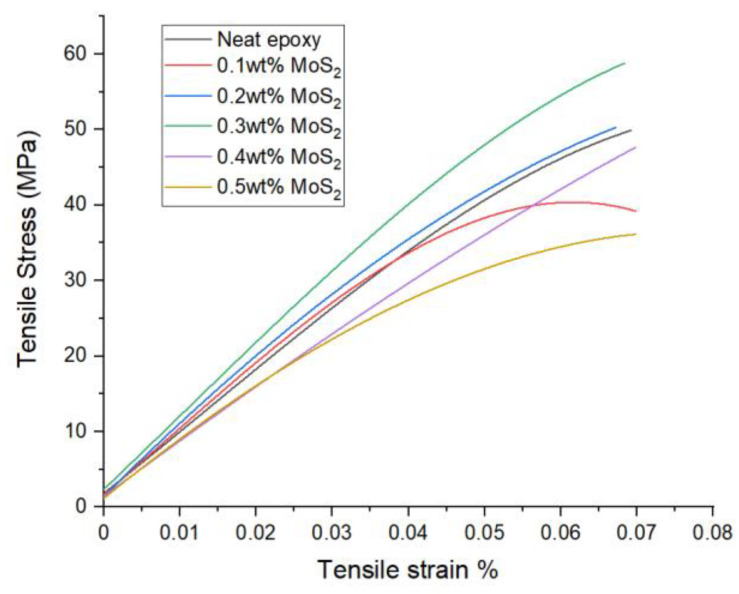
Tensile stress–strain curves of neat epoxy and epoxy composite with different MoS_2_ loading.

Figure 3 illustrates the stress versus strain curve of both neat epoxy and composites consisting of 0.1 wt% to 0.5 wt% MoS_2_/epoxy. The graph illustrates the characteristic ductility of epoxy material. The incorporation of MoS_2_ facilitated efficient stress transfer through interlayer hydrogen bonding and intra-layer interactions. However, it was observed that the addition of 0.1 wt% MoS_2_/epoxy resulted in lower tensile and Young’s modulus values (approximately 9%) compared to neat epoxy. This phenomenon can be attributed to MoS_2_ potentially obstructing the nitrogen groups in the amine curing agent’s reaction with the epoxy polymer resin, consequently leading to a slight decrement in the overall tensile properties of the composites. This observation suggests that some of the cross-links may have been replaced by linear interactions between the nanofiller and epoxy chains instead of the polyamine hardener. Additionally, this could be linked to the intrinsic hydrophobic nature of MoS_2_ [13]. With the 0.1 wt% MoS_2_ filler content, there is a notable increase in the hardness of the composite, signifying a higher degree of curing and crosslinking. Alternatively, it is worth noting that low crosslink density tends to decrease hardness.

### 3.3. Friction and Wear Properties

We employed 0.3 wt% MoS_2_/epoxy composites for all tribological testing since they exhibited the optimized mechanical properties. Surface profilometry was employed to measure the surface roughness of the polished epoxy and 0.3 wt% MoS_2_ composites. The epoxy displayed an Rq value of 1.8 µm, while the MoS_2_/epoxy composites containing 0.3 wt% MoS_2_ exhibited Rq values of 0.4 µm. These results suggest that MoS_2_ content leads to smoother surface topography in the epoxy matrix during mechanical polishing. The tribological properties of both epoxy and 0.3 wt% MoS_2_/epoxy composites were investigated under various test conditions using linear reciprocating tribological testing. The specimens were slid against a 10 mm steel ball for a total distance of 400 m at different sliding speeds (0.02 ms^−1^, 0.07 ms^−1^, and 0.2 ms^−1^) and under varying normal loads (5 N, 10 N, and 15 N), and results are summarized in Table 2.

**Figure 4:**
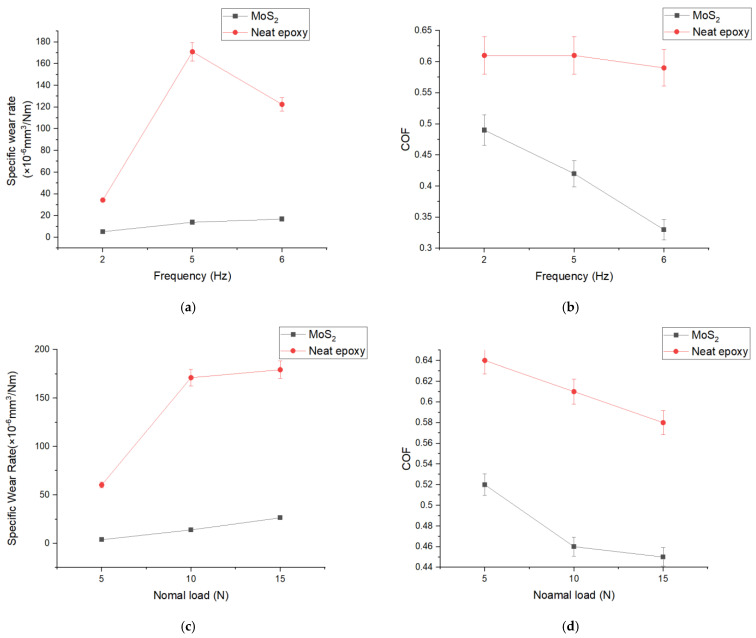
Neat epoxy and 0.3 wt% MoS_2_/epoxy of composite under 2 Hz, 5 Hz, and 8 Hz with a constant normal load of 10 N; (**a**) Specific wear rate; (**b**) Average friction coefficient; and under 5 N, 10 N, and 15 N applied loads with constant 5 Hz frequency; (**c**) Specific wear rate; (**d**) Average friction coefficient.

**Figure 5:**
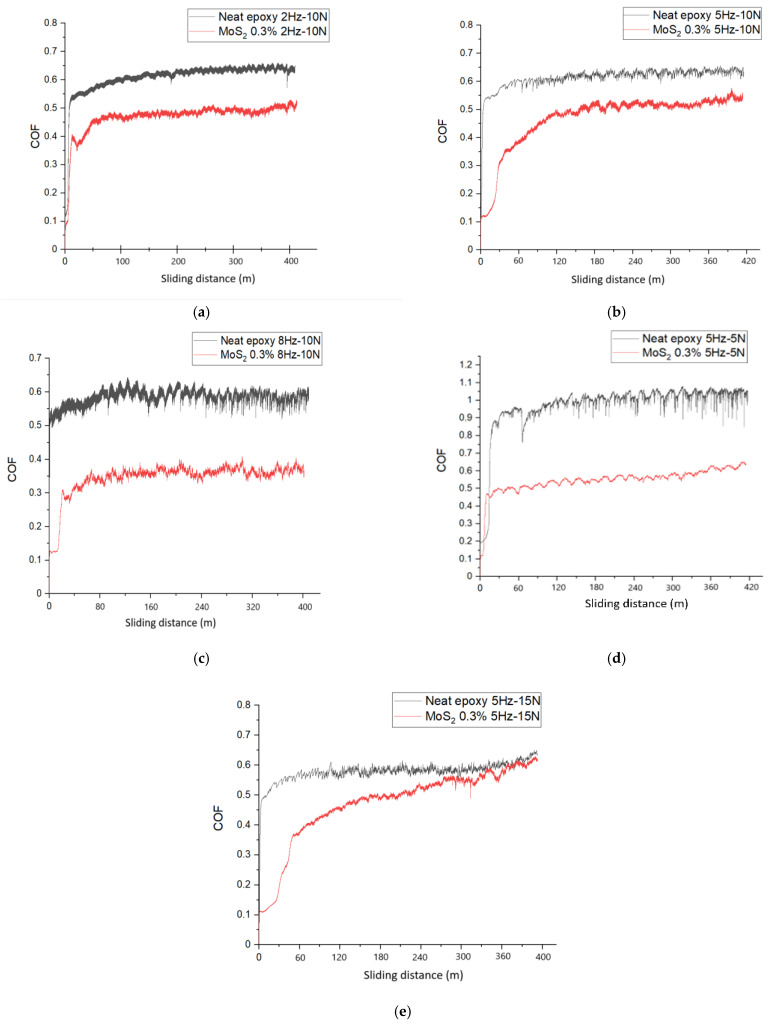
Variation of COF versus sliding distance of neat epoxy and 0.3%wt%MoS_2_/epoxy composite under different test conditions: (**a**) 2 Hz/10 N; (**b**) 5 Hz/10 N; (**c**) 8 Hz/10 N; (**d**) 5 Hz/5 N; and (**e**) 5 Hz/15 N.

Figure 4a depicts the specific wear rates of the 0.3 wt% MoS_2_/epoxy composite and neat epoxy under different sliding speeds of 0.02 ms^−1^, 0.07 ms^−1^, and 0.2 ms^−1^ (2 Hz, 5 Hz, and 8 Hz) at a constant normal load of 10 N. For neat epoxy, the specific wear rates were measured at 34.3 × 10^−6^, 171.0 × 10^−6^, and 122.5 × 10^−6^ mm^3^/N·m for 0.02 ms^−1^, 0.07 ms^−1^, and 0.2 ms^−1^ speeds, respectively. In contrast, the incorporation of 0.3 wt% MoS_2_ as a filler significantly reduced the specific wear rate of the epoxy to 5.2 × 10^−6^, 13.9 × 10^−6^, and 16.8 × 10^−6^ mm^3^/N.m. This represents a decrease of specific wear rate by 84.84%, 91.87%, and 86.29% compared to neat epoxy. Figure 4b illustrates the average COF of both the 0.3 wt% MoS_2_/epoxy composite and neat epoxy under varying sliding speeds at a constant normal load of 10 N. In the case of neat epoxy, the COF was measured at 0.61, 0.61, and 0.59 for speeds of 0.02 ms^−1^, 0.07 ms^−1^, and 0.2 ms^−1^, respectively. Conversely, with the inclusion of 0.3 wt% MoS_2_ as a filler, the COF of the epoxy was notably reduced to 0.49, 0.42, and 0.33, respectively. This indicates an improvement of 19.67%, 31.15%, and 44.07% compared to the COF of neat epoxy. These findings suggest that MoS_2_ sheets contribute to the decreased friction in the epoxy composite by acting as a solid lubricant for the contact interface. Additionally, MoS_2_ can serve as spacers, preventing direct contact between the steel ball and the composite. Another noteworthy observation is that with an increase in sliding speed, the wear rate of the 0.3 wt% MoS_2_/epoxy significantly increases from 5.2 × 10^−6^ to 16.8 × 10^−6^ mm^3^/N.m, while the COF decreases from 0.49 to 0.33. Typically, increasing the speed of sliding leads to an increase in the temperature on the sliding surface, rendering the material softer. Consequently, this contributes to an increase in the wear volume of the composite. Consequently, the volume of MoS_2_ contributing to friction reduction exceeds the lower wear rate, thereby gradually reducing the COF. This observation can be further supported by the adhesive wear observed in SEM images in Figure 5.

Figure 4d presents the average COF for both the 0.3 wt% MoS_2_/epoxy composite and neat epoxy under varying normal loads (5 N, 10 N, and 15 N) at a sliding frequency of 5 Hz. For neat epoxy, the COF values were recorded as 0.64, 0.61, and 0.58 for normal loads of 5 N, 10 N, and 15 N, respectively. Conversely, with the addition of 0.3 wt% MoS_2_ as a filler, the COF of the epoxy notably decreased to 0.52, 0.46, and 0.45, respectively. This signifies a reduction of 18.75%, 24.59%, and 22.41% compared to the COF of neat epoxy. Figure 4c illustrates the specific wear rates of both the 0.3 wt% MoS_2_/epoxy composite and neat epoxy under different normal loads (5 N, 10 N, and 15 N) at a constant sliding frequency of 5 Hz. For neat epoxy, the specific wear rates were measured at 60.3 × 10^−6^, 171.1 × 10^−6^, and 179.3 × 10^−6^ mm^3^/N.m for normal loads of 5 N, 10 N, and 15 N, respectively. In contrast, the incorporation of 0.3 wt% MoS_2_ as a filler significantly reduced the specific wear rate of the epoxy to 3.8 × 10^−6^, 13.9 × 10^−6^, and 26.4 × 10^−6^ mm^3^/Nm. This represents a significant decrease in specific wear rate by 93.70%, 91.88%, and 85.28% compared to neat epoxy.

To enhance the wear resistance of polymers, the addition of MoS_2_ particles offers several advantages. Firstly, MoS_2_ is believed to blend effectively with wear particles, strengthening the transferred film and acting as a solid lubricant. Secondly, MoS_2_ enhances the mechanical strength of the epoxy matrix, enabling it to withstand high sliding loads. Additionally, the uniform dispersion of MoS_2_ in the epoxy matrix facilitates efficient release of MoS_2_ from the microstructure during sliding, effectively incorporating MoS_2_ into the tribofilm and reducing friction even at low concentrations of MoS_2_ filler. The influence of surface roughness on the friction of epoxy composites is significant, as rougher surfaces can generate higher friction through mechanical interlocking between surface asperities. Therefore, the reduction in surface roughness of epoxy composites with increased MoS_2_ content may contribute to decreased friction. Furthermore, higher wear volume resulting in larger contact between the steel ball and composite can lead to increased friction during sliding contact. However, the increased elastic modulus of epoxy composites with MoS_2_ content decreases friction by reducing their contact area with the steel ball.

The introduction of MoS_2_ content leads to a noticeable reduction in the friction coefficient of epoxy composites throughout the entire sliding process, indicating that MoS_2_-modified epoxy composites exhibit lower friction compared to pure epoxy. During sliding, a transfer film or layer forms on the surface of the epoxy composite as it interacts with the steel counterpart. This film transfer reduces metallic contact between the sample and the steel ball, consequently decreasing the wear rate. Under lower normal load conditions, the wear rate demonstrates lower values, with the wear mechanism characterized by mild adhesive wear. However, with increasing applied load, fatigue and abrasive wear occur, resulting in the formation of a thicker transfer film. Thus, a thicker transfer layer is formed under higher loads. To further demonstrate the idea, we performed SEM observations of the wear surface topology and wear track cross-section.

### 3.4. Effect of MoS_2_ on Wear and Transfer Film

It is widely acknowledged that the interaction between epoxy and chromium steel surfaces results in notable wear [13]. This phenomenon occurs due to the generation of fragmented epoxy during sliding, accompanied by the absence of a transfer film. However, by incorporating inorganic material MoS_2_ into the epoxy, a cohesive film of the filled composite, several microns in thickness, is deposited onto the counter face. As a result, the specific wear rate and COF are reduced in comparison to those observed with the unfilled material. The effectiveness of the MoS_2_ on tribo-layer development and the role of MoS_2_ in COF are well illustrated in Figure 5 with different test conditions. It shows that the MoS_2_ as a filler in epoxy reduced the COF of epoxy in sliding against the chromium steel ball. Unfilled neat epoxy is unable to form a continuous film.

Figure 5a demonstrates the variation of the COF versus the sliding distance (400 m) during a 2 Hz/10 N wear test on both unfilled neat epoxy and the MoS_2_/epoxy composite. Similarly, Figure 5b,c present the COF over the sliding distance during wear tests at increased sliding frequencies of 5 Hz and 8 Hz at constant 10 N load, respectively, on both unfilled neat epoxy and the MoS_2_/epoxy composite. The findings demonstrate that at 2 Hz/10 N test conditions, epoxy and MoS_2_/epoxy composites exhibit a stable average COF value of 6.1 and 4.9, respectively. Figure 5b,d,e present the COF variation during wear tests at different normal loads of 5 N, 10 N, and 15 N at a constant 5 Hz sliding frequency on both unfilled neat epoxy and the MoS_2_/epoxy composite. The test condition of 8 Hz/10 N gives the stable low value of COF around 3.3 with the time over the 400 m total sliding distance.

**Figure 6:**
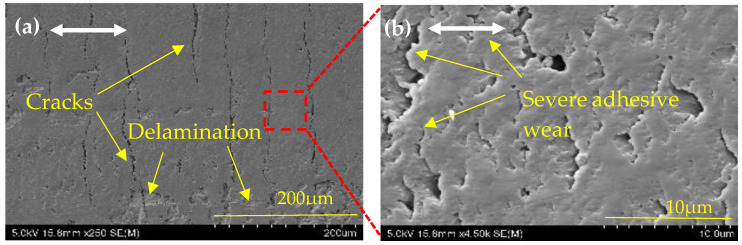

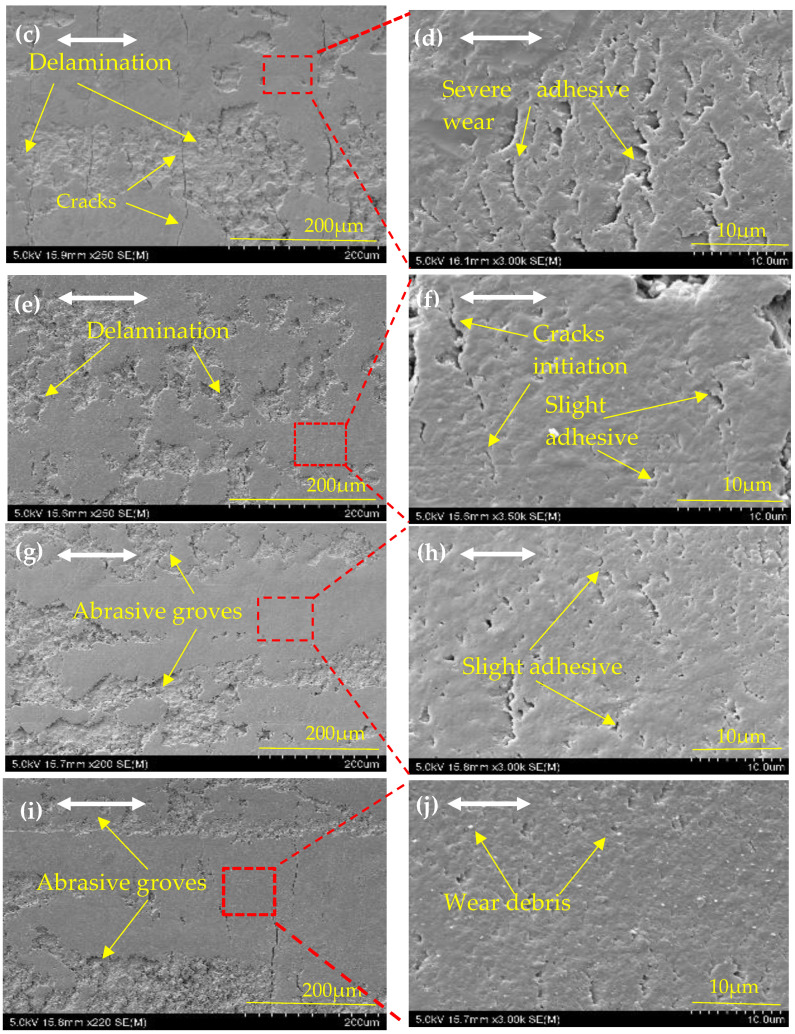
The topology of different magnifications of SEM images of the wear track of neat epoxy under different test conditions: (**a**,**b**) 2 Hz/10 N; (**c**,**d**) 5 Hz/10 N; (**e**,**f**) 8 Hz/10 N; (**g**,**h**) 5 Hz/5 N; (**i**,**j**) 5 Hz/15 N. A two-way arrow shows the linear reciprocating sliding direction.

**Figure 7:**
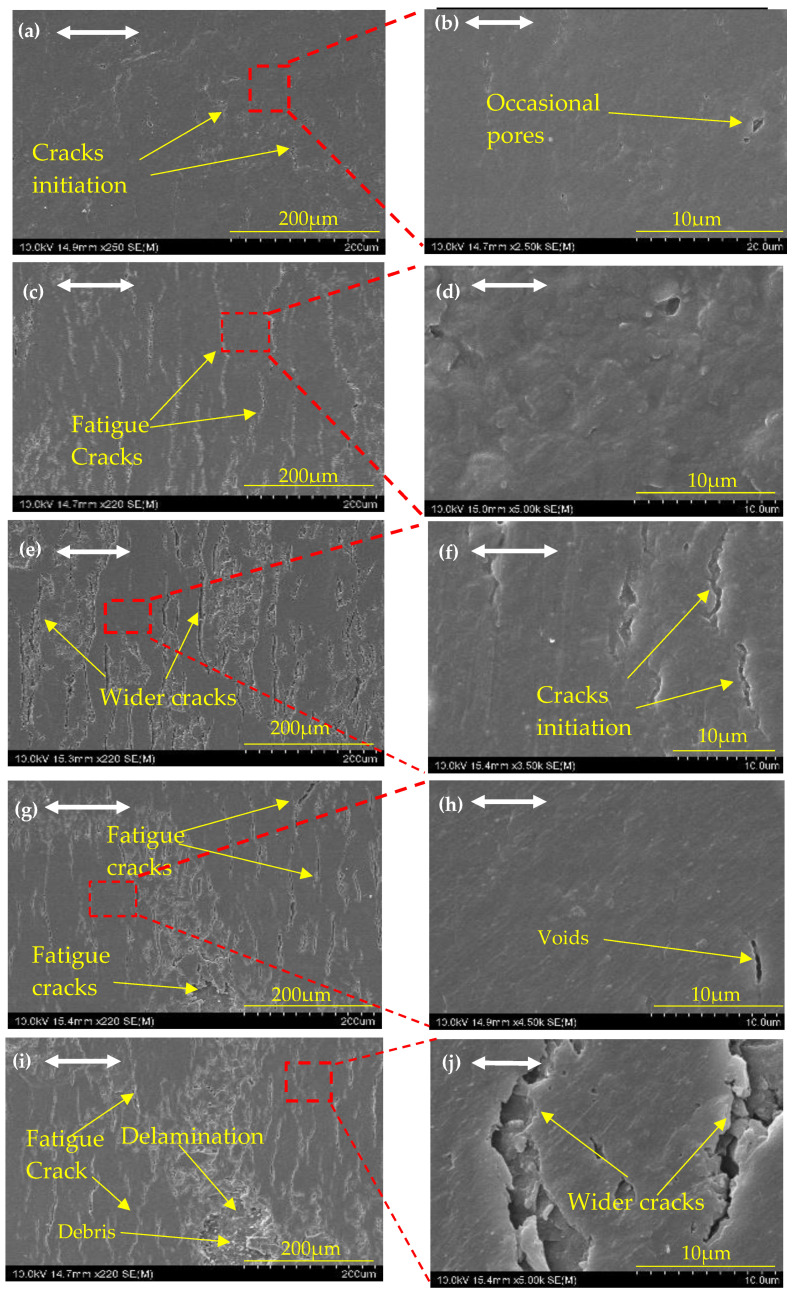
The topology of different magnifications of SEM images of the wear track of MoS_2_/epoxy under different test conditions: (**a**,**b**) 2 Hz/10 N; (**c**,**d**) 5 Hz/10 N; (**e**,**f**) 8 Hz/10 N; (**g**,**h**) 5 Hz/5 N; (**i**,**j**) 5 Hz/15 N. A two-way arrow shows the linear reciprocating sliding direction.

Figure 6a,b show SEM images at various magnifications of the wear surfaces of neat epoxy composites under the 2 Hz/10 N test condition. It reveals the presence of numerous cracks perpendicular to the sliding axis on the wear surface, indicative of fatigue crack development over time. Furthermore, at higher magnification, significant adhesive wear features can be seen. This is caused by thermal softening at the sliding interface due to epoxy’s poor heat transfer properties [13]. Corresponding SEM images of 0.3 wt% MoS_2_/epoxy composite at different magnifications of the wear surfaces under the 5 Hz/10 N, 8 Hz/10 N, 5 Hz/5 N, and 5 Hz/15 N are depicted in Figure 7. In contrast, the corresponding MoS_2_/epoxy composite exhibits a relatively smooth and uniform wear surface with occasional pores in Figure 7a,b. This morphology minimizes the occurrence of polymer adhesive wear during sliding. Added MoS_2_ greatly reduced the adhesive wear during sliding. When the sliding frequencies of the neat epoxy wear test are increased to 5 Hz and 8 Hz under a constant 10 N, Figure 6c,e show evidence of extensive adhesive wear and the delamination of materials from the neat epoxy surface. Corresponding MoS_2_/epoxy composites in Figure 7c,e show cracks propagating perpendicular to the wear axis but under high magnification, showing the smoothness of the surface and still mitigating the adhesive wear. Despite this observation, the COF values of MoS_2_/epoxy composite remain low and stable around 0.33 even when increasing the sliding speed from 0.02 ms^−1^ to 0.2 ms^−1^. Figure 6e displays adhesive debris pulled off from the softer surface. Throughout the sliding process, epoxy surface irregularities experience substantial fracturing or plastic deformation. The subsurface undergoes strain hardening and plastic deformation as well. The SEM micrograph of the worn-out surface reveals the extensive pull-off material accompanied by plastic deformation. Figure 6g,i depict profound surface structural changes consistent with severe wear. This severe form of adhesion wear is also known as smearing, galling, and scuffing.

SEM images of neat epoxy at different magnifications of the wear surfaces under the wear test conditions for 5 N/5 Hz, 10 N/5 Hz, and 15 N/5 Hz are shown in Figure 6d and 6g and 6c and 6i, respectively. When increasing the normal load of the wear test up to 10 N and 15 N, the abrasive wear grooves appeared on the neat epoxy wear surface of Figure 6g,i. When increasing the normal load to 10 N and 15 N, the abrasive wear groves appeared on the neat epoxy wear surface consisting of rod-shaped wear debris. This characteristic indicates that abrasive wear dominates over adhesive wear, resulting in a notably higher specific wear rate of 179.3 ± 8.965 × 10^−6^ mm^3^/N·m, which stands as the highest reported value during these experiments. The corresponding MoS_2_/epoxy composite in Figure 7c,g shows the micro-cracks and delamination.

**Figure 8:**
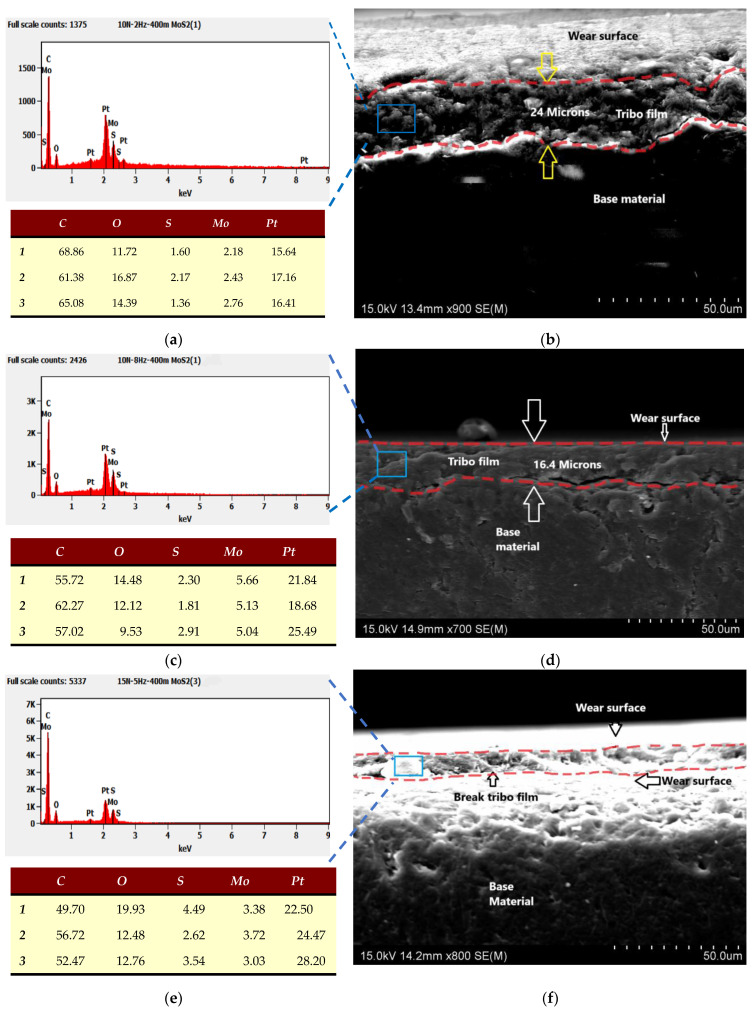
EDS analysis of chemical composition and corresponding SEM images of cross-sections of wear tracks of 0.3%wt%MoS_2_/epoxy (**a**,**b**) for 2 Hz/10 N; (**c**,**d**) for 8 Hz/10 N; (**e**,**f**) for 5 Hz/5 N.

To gain a clearer understanding of the tribofilm and the wear mechanism, the cross-section of the wear track was examined under SEM for both neat epoxy and MoS_2_/epoxy composite test pieces. This analysis aims to delve deeper into the composition of the tribofilm on the wear surface of the material as shown in Figure 8. Figure 8a illustrates the EDS analysis of the chemical composition of the wear surface of the MoS_2_/epoxy composite tested under 2 Hz/10 N conditions. Figure 8b,c show the corresponding wear track cross-section SEM images of MoS_2_/epoxy composite and neat epoxy.

Figure 8a EDS analysis reveals the average MoS_2_ content on the top of the wear track was 2.46 wt% under 2 Hz/10 N conditions, whereas the bulk materials consist of 0.3 wt% of MoS_2_ content. This finding suggests that MoS_2_ is transferred from the epoxy matrix to the wear surface during sliding, contributing to the formation of a thin tribofilm enriched with MoS_2_ atop the track (Figure 8b). This tribofilm has a thickness of approximately 26–22 microns and facilitates lubrication during sliding with the steel counterpart, resulting in a low COF value. When the sliding frequency of the wear test is increased to 8 Hz, Figure 8c shows the EDS results of the MoS_2_/epoxy composite wear surface, indicating a higher average MoS_2_ content of 5.27 wt%. This suggested a thicker and more stable tribofilm, which is consistent with the observed lowest and most stable COF value. The corresponding Figure 8d shows a wear track cross-section with a relatively thin tribofilm (13–21 microns) on the surface.

The addition of MoS_2_ significantly enhances the resistance of epoxy against abrasive wear. The evolution of the COF over time during a 5 Hz/15 N wear test is illustrated in Figure 5e. While the MoS_2_/epoxy composite initially exhibits a lower COF value, it shows an increasing trend over time, eventually aligning with the COF value of neat epoxy.

The corresponding SEM image Figure 7i of the MoS_2_/epoxy composite reveals notable cracks and surface breakage over time. This observation sheds light on the formation and eventual breakdown of the tribofilm on the wear track surface under 15 N normal loads. Additionally, Figure 8e presents the corresponding energy-dispersive EDS results of the MoS_2_/epoxy composite wear surface at 5 Hz/15 N, indicating an average 3.35 wt% of Mo. Furthermore, Figure 8f displays a cross-sectional SEM image showing the breakage of the thin film over the wear surface, leading to direct contact between the steel ball and the material surface.

**Figure 9:**
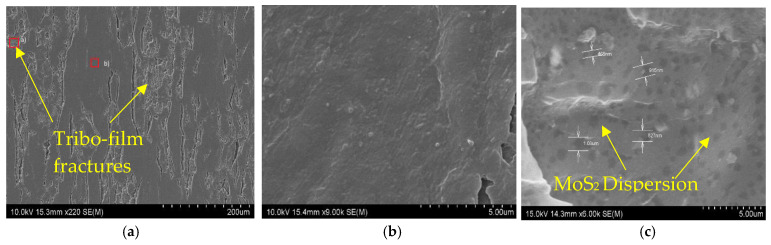
SEM micrographs of the wear track for 0.3 wt% MoS_2_/epoxy composite at 8 Hz/10 N with (**a**) lower magnification; (**b**) MoS_2_ dispersion through the tribo-film fracture and; (**c**) smooth surface of the tribo-film under higher magnification.

**Figure 10:**
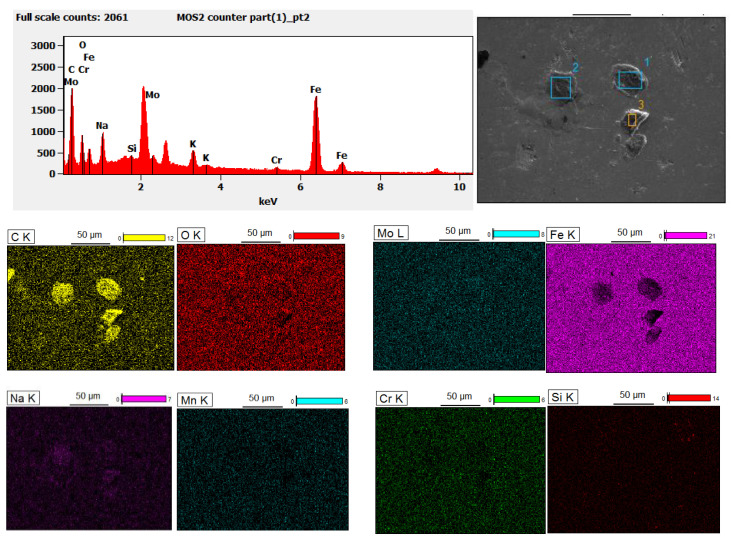
EDS analysis of the chemical composition of the transfer layer on the counter steel ball and corresponding SEM image. EDS analysis is correlated with the number 2 area of the SEM image.

Figure 9a presents SEM images of the wear surface of the MoS_2_/epoxy composite corresponding to the 10 N/8 Hz test condition, which yielded the lowest and most stable COF value of 0.33. In Figure 9a, the wear surface is depicted, while Figure 9b,c show the surfaces of the thin film and the fracture surface of the MoS_2_/epoxy composite under the 10 N/8 Hz sliding test, respectively. The fracture surface shown in Figure 9b displays a uniform distribution of exfoliated MoS_2_ within the epoxy matrix, with a typical flake size distribution ranging from 0.35 to 1.5 microns. This observation underscores the effectiveness of ultrasound exfoliation in dispersing MoS_2_ within the epoxy resin and its role in forming a relatively high concentration of MoS_2_ in the tribofilm. Figure 9c illustrates the top surface SEM observation of the thin film formed during dry sliding, revealing an average MoS_2_ content of 5.27 wt%. This image further demonstrates how, during sliding, MoS_2_ becomes firmly integrated with the epoxy wear debris by indicating the absence of distinct MoS_2_ flakes. Figure 10 illustrates the SEM image of the transfer layer on the surface of the counter steel ball, corresponding to the 10 N/8 Hz sliding test, along with the EDS analysis of the chemical composition of the wear surface of the steel ball. The results indicate that the transfer layer from the composite to the surface of the counter steel ball contains 32.11 to 42.37 wt% of carbon and 1.03 to 2.34 wt% of Mo, uniformly distributed across the wear surface of the steel ball. It can be concluded that the generated tribofilm was acting as a spacer between the epoxy composite and the steel counter ball.

## 4. Conclusions

The study delved into the systematic investigation of the effects of ultrasonication exfoliated MoS_2_, ranging from 0.1 to 0.5 wt%, on the mechanical and tribological properties of epoxy composites. To enhance the wear resistance of polymers, the addition of MoS_2_ particles offers several advantages. Firstly, MoS_2_ is believed to blend effectively with wear particles, strengthening the transferred film and acting as a solid lubricant. Secondly, MoS_2_ enhances the mechanical strength of the epoxy matrix, enabling it to withstand high sliding loads. Additionally, the uniform dispersion of MoS_2_ in the epoxy matrix facilitates efficient removal of MoS_2_ from the microstructure during sliding, effectively incorporating MoS_2_ into the tribofilm and reducing friction even at ultra-low concentrations of MoS_2_ filler.

Optimized mechanical properties of the MoS_2_/epoxy composite were achieved at 0.3 wt% MoS_2_ content with the epoxy resin. Specifically, the resulting composite exhibited a hardness of 88.0 Shore D, an ultimate tensile strength of 61.88 MPa, and a modulus of elasticity of 0.86 GPa, indicating increments of 61.5%, 35.45%, and 16.21%, respectively. Subsequently, the 3 wt% MoS_2_/epoxy composite, possessing the highest MoS_2_ composition, underwent tribological property examination. Two series of tests were conducted: firstly, to assess wear rate and COF with an increase in sliding frequency from 2 Hz to 8 Hz while maintaining a normal load of 10 N and a sliding distance of 400 m; secondly, to evaluate the effects of increasing the normal load from 5 N to 15 N while keeping the sliding frequency at 5 Hz and the sliding distance constant. The lowest mean COF value of 0.33 was obtained with the 10 N/8 Hz MoS_2_/epoxy test, representing a 44.07% reduction compared to neat epoxy, while the corresponding wear rate was 16.8 × 10^−6^ mm^3^/N·m, indicating an 86.29% reduction compared to neat epoxy.

The high sliding frequency of 8 Hz at higher accelerates the frequent stretching of the material’s bare surface, particularly in the initial stages, aiding in the efficient removal of MoS_2_ from the epoxy matrix and increasing the amount of MoS_2_ content to 5.04 wt% in the tribofilm. The corresponding observation is provided by which displays EDS analysis of the test piece subjected to a wear test condition of 2 Hz/10 N, revealing a low MoS_2_ 0.3 wt% at low wear speeds. Consequently, the weight fraction of MoS_2_ in the generated tribofilm is notably low. Despite utilizing a considerably low amount of MoS_2_ weight fraction, it significantly contributes to the formation of effective tribofilm. The initial exfoliation of the MoS_2_ flakes increases their surface area, and subsequent ultrasonication of the blend of epoxy/MoS_2_ effectively disperses the MoS_2_ sheets within the epoxy matrix. The uniform distribution of an extremely low amount of filler (0.3 wt%) enhances the likelihood of its presence on the rubbing surface, where the high-speed steel ball effectively removes the MoS_2_. Ultimately, this high fraction of MoS_2_ blended with the wear debris of epoxy forms a stable and uniform tribofilm on the wear surface. Moreover, MoS_2_ is a well-known solid lubricant that lubricates between the epoxy test piece and the counter steel ball, reducing both wear and COF values. These observations demonstrate that even an ultralow amount of MoS_2_ is adequate for effective lubrication in epoxy polymer, provided there are extra precautions taken for uniform particle dispersion during mixing with the resin. It could be concluded that the incorporation of a low amount of MoS_2_ could have a significant effect on the mechanical and tribological properties of the epoxy composites with ultrasonication mixing. Future research could explore and investigate the long-term durability and environmental stability of MoS_2_ thin films under varying operational conditions. Specifically, studies could examine the effects of exposure to atmospheric oxygen and humidity, which may lead to the formation of MoO_3_ and negatively impact lubrication performance. Understanding these factors will provide critical insights into the reliability and longevity of MoS_2_-based composites in real-world applications.

## Figures and Tables

**Table 1:** Summary of the mechanical test properties.

Composite System	Elongation at Break	Ultimate Tensile Strength (MPa)	Young’s Modulus (GPa)	Hardness (Shore D)
Neat Epoxy	0.064 ± 0.0035	45.683 ± 2.37	0.74037 ± 0.022	53 ± 1.59
0.1 wt% MoS_2_	0.066 ± 0.0032	41.358 ± 2.24	0.64528 ± 0.019	75 ± 2.25
0.2 wt% MoS_2_	0.062 ± 0.0036	44.128 ± 2.32	0.75434 ± 0.022	85 ± 2.55
0.3 wt% MoS_2_	0.065 ± 0.0034	61.880 ± 2.85	0.85973 ± 0.025	88 ± 2.64
0.4 wt% MoS_2_	0.085 ± 0.0025	53.333 ± 2.60	0.81807 ± 0.024	81 ± 2.43
0.5 wt% MoS_2_	0.083 ± 0.0035	38.615 ± 2.15	0.66297 ± 0.019	76 ± 2.28

**Table 2:** Summary of the tribological test properties.

At Constant Load (10 N)	Specific Wear Rate (×10^−6^ mm^3^/N.m) MoS_2_ 0.3 wt%	Specific Wear Rate (×10^−6^ mm^3^/N.m) Neat Epoxy	COF MoS_2_ 0.3 wt%	COF Neat Epoxy
2 Hz	05.2 ± 0.260	034.3 ± 1.715	0.49 ± 0.0245	0.61 ± 0.0305
5 Hz	13.9 ± 0.695	171.0 ± 8.550	0.42 ± 0.021	0.61 ± 0.0305
8 Hz	16.8 ± 0.840	122.5 ± 6.125	0.33 ± 0.0165	0.59 ± 0.0295
**At Constant Frequencies** **(5 Hz)**	**Specific Wear Rate** **(×10^−6^ mm^3^/N.m)** **MoS_2_ 0.3 wt%**	**Specific Wear Rate** **(×10^−6^ mm^3^/N.m)** **Neat Epoxy**	**COF** **MoS_2_ 0.3 wt%**	**COF** **Neat Epoxy**
5 N	03.8 ± 0.190	060.3 ± 3.015	0.52 ± 0.026	0.64 ± 0.032
10 N	13.9 ± 0.695	171.1 ± 8.555	0.46 ± 0.023	0.61 ± 0.031
15 N	26.4 ± 1.320	179.3 ± 8.965	0.45 ± 0.023	0.58 ± 0.029

## Data Availability

Data presented in this study are available upon request.
